# Computational drug repurposing for inflammatory bowel disease using genetic information

**DOI:** 10.1016/j.csbj.2019.01.001

**Published:** 2019-01-07

**Authors:** Liam Grenier, Pingzhao Hu

**Affiliations:** a Department of Biochemistry and Medical Genetics and The George and Fay Yee Centre for Healthcare Innovation, University of Manitoba, Winnipeg, MB, Canada; b Department of Electrical and Computer Engineering, University of Manitoba, Winnipeg, MB, Canada

**Keywords:** Inflammatory bowel disease, Risk genes, Drug repurposing, Computational approach, Pathway enrichment

## Abstract

As knowledge of the genetics behind inflammatory bowel disease (IBD) has continually improved, there has been a demand for methods that can use this data in a clinically significant way. Genome-wide association analyses for IBD have identified 232 risk genetic loci for the disorder. While identification of these risk loci enriches our understanding of the underlying biology of the disorder, their identification does not serve a clinical purpose. A potential use of this genetic information is to look for potential IBD drugs that target these loci in a procedure known as drug repurposing. The demand for new drug treatments for IBD is high due to the side effects and high costs of current treatments. We hypothesize that IBD genetic variants obtained from GWAS and the candidate genes prioritized from the variants have a causal relationship with IBD drug targets. A computational drug repositioning study was done due to its efficiency and inexpensiveness compared to traditional in vitro or biochemical approaches. Our approach for drug repurposing was multi-layered; it not only focused on the interactions between drugs and risk IBD genes, but also the interactions between drugs and all of the biological pathways the risk genes are involved in. We prioritized IBD candidate genes using identified genetic variants and identified potential drug targets and drugs that can be potentially repositioned or developed for IBD using the identified candidate genes. Our analysis strategy can be applied to repurpose drugs for other complex diseases using their risk genes identified from genetic analysis.

## Graphical abstract

**Figure:**
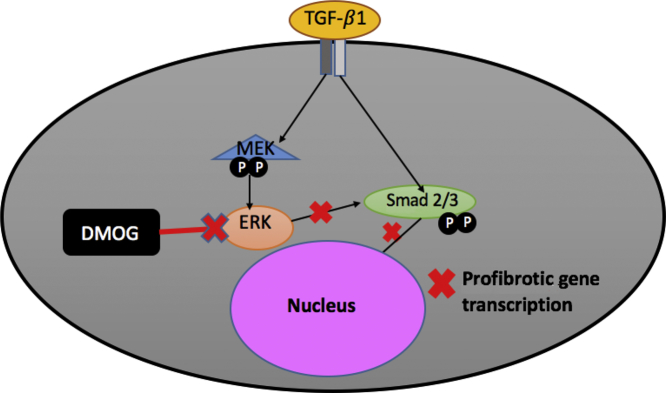

## 1 Introduction

Inflammatory bowel disease (IBD) is a chronic, debilitating disorder characterized by the inflammation of the intestines. The disorder is prevalent worldwide and its incidence is continually increasing [1]. Symptoms of the disorder range from mild to severe and include abdominal pain, intestinal bleeding, weight loss, fever, and diarrhea [2]. The two subtypes of the disorder are Crohn's disease (CD) and ulcerative colitis (UC). CD can affect any portion of the GI tract, while UC is confined to the colon. UC is confined to the colon. The chronic inflammation that characterizes IBD is believed to be due to the dysregulation of the innate and adaptive immune responses [3]. The exact etiology of the disease is not fully understood, but a combination of interactions between genetic, environmental, and microbial factors with the immune system is thought to underlie the disorder [3].

The continual expansion of our knowledge on the genetic architecture of IBD is allowing for the potential of finding new targeted therapy for the disorder [4]. The genetic risk variants for IBD identified are the main source for the potential of new targeted treatments. The finding of these variants will improve clinical practices for IBD in three ways: 1) Better understanding of disease mechanism allows for the development of targeted therapies; 2) Permits the prediction of disease in the general population which allows for preventative measures; and 3) allows for the adapting of medical therapy based on the genetic profile of a single patient [4]. The development of personalized medicine is one of the main possible benefits from understanding genetic risk variants. It would allow clinicians to choose the best treatment for a patient based on the severity of their condition, as the disease course of IBD is variable. This would circumvent the use of debilitating drugs with severe side effects on patients with milder forms of the disease and allow immediate use of more aggressive treatments in those with more serious forms of the disease [4]. The challenges faced in using genetic information for IBD is identifying specific genes in these risk gene regions that are the causal variant. Once a causal gene is identified, its function must be well known, and the protein that the gene encodes must then be able to be targeted with a drug without causing severe side effects in the target [4]. It would also be difficult to screen individuals at increased risk of developing IBD based solely on genetic background due to the fact that relatively moderate role genetic factors have in IBD development, and environmental factors, lifestyle and diet, and the microbiome of the gut are significant determinants of IBD development that are not well understood.

The establishment of genome-wide association studies (GWAS) has led to the advancement of our understanding with regards to complex diseases with a strong genetic component such as IBD. GWAS analyze DNA sequence variations (single nucleotide polymorphism (SNP)) from across the human genome to identify genetic risk variants for disease common in the population [5]. GWAS treat SNPs as genetic markers that are used to map genomic regions. SNPs are generally not phenotypically harmful. However, certain SNPs can be disease-causing due to changes of amino acid, mRNA transcript stability, and transcription factor binding affinity [5]. GWAS basically compare the genomes (usually collected using microarray analysis or next generation sequencing (NGS)) of a control population and a population with a certain disorder of interest to determine whether there are SNPs that are more frequently present in the disease population compared to the control. Several GWAS for IBD have been performed throughout the past few years and have yielded 232 IBD associated SNPs [6].

In terms of treatment, current IBD drugs aim to induce deep remission of symptoms. Older treatments of IBD only aimed to maintain remission of symptoms. Several classes of drugs are used, which are highlighted in Table 1.

**Table 1:** Current IBD drugs.

Drug	Drug type	Drug function
Mesalazine, Olsalazine, Sulfasalazine, Balsalazide	Aminosalicylates [7]	Anti-Inflammatory (for mild to moderate IBD)
Prednisolone, Prednisone, Hydrocortisone, Methylprednisolone, Budesonide, Desonide, Betamethasone, Triamcinolone, Dexamethasone, Cortisone Acetate	Corticosteroids [7]	Anti-Inflammatory (for moderate to severe IBD)
Azathioprine, 6-Mercaptopurine, Methotrexate	Thiopurines [7]	Immunosuppression (for aminosalicylate resistant patients with chronic corticosteroid use)
Ciprofloxacin, Metronidazole,	Antibiotics [7]	Modulation of gut Microbiota Treatment of bacterial infections
Infliximab, Adalimumab, Certolizumab, Golumumab	TNF blockers [7]	Anti-inflammatory through down regulation of TNF signaling (for moderate to severe IBD in patients resistant to other treatments)
Natalizumab, Vedolizumab	Leucocyte adhesion inhibitors [7]	Anti-inflammatory through preventing white blood cells from adhering to blood vessels and entering target tissue. (for CD patients whose disease is resistant to all other therapies)
Ustekinumab	Anti-Interleukin-12/23 [8]	Prevent the action of proinflammatory cytokines IL-12 and IL-23 in CD. Used in patients with moderate to severe CD
Tofacitinib	JAK inhibitor [8]	Inhibits JAK/STAT signaling pathway which prevents transcription of multiple inflammatory cytokines involved in IBD pathogenesis. Approved for us in patients with UC

There are two general approaches to disease treatment: the top-down and step-up approaches [4]. The top-down approach is most commonly used in IBD; it involves the early administration of biological therapies in the disease course. This approach can be problematic as it leads to over-zealous treatment of many patients. This gives potential to step-up approaches that use milder treatments at the beginning of treatment with the option of escalating treatment if the initial therapy does not work. The hope is, once personalized medicine is a feasible treatment route, these two approaches will become obsolete; each patient would receive treatment tailored specifically to their condition.

The problem with current IBD drugs are their numerous side effects, and their tendencies to stop being effective after long term use (patients develop tolerance to the drugs over time). This is associated with a negative quality of life in those with IBD. Besides the symptoms of the disease and the side effect of drug treatments, patients are also negatively impacted by the high costs of the medications they are using, further strengthening the need to identify new drugs for the disease.

The need for new drugs in IBD treatment has brought attention to the procedure known as drug repurposing. The goal of a repurposing study is to find new uses for existing drugs. In our case, the aim was to find drugs to target IBD (that weren't used for IBD currently). Two common approaches for drug repurposing exist: in vivo or biochemical experiments and computational methods. Computational methods are more efficient and less costly compared to biochemical methods. This approach predicts drug-target interactions to identify new drugs or drug targets for a given disease of interest. Traditional drug development is extremely costly, which takes 10–15 years to develop a drug. Only around 20 new drugs are developed each year [9]. The high cost is usually attributed to developed drugs that fail testing in clinical trials due to having unexpected toxicity or lack of efficacy. Drug candidates obtained through this procedure can then be validated in vivo. The efficient and inexpensive qualities of computational methods made it the drug repurposing procedure of choice [4,10]. The substantial genetic data available on IBD was taken advantage of to perform the drug repurposing analysis. We hypothesize that focusing on drugs that target the genetic risk regions associated with IBD can speed up the identification of suitable drug candidates. This strategy is relatively young but is believed to become an important tool for drug development [4]. Candidate IBD drugs were thus based on drugs that could significantly target proteins coded by identified IBD risk genes and also target proteins that interact with these encoded proteins in biological pathways.

## 2 Material and Methods

**Fig. 1:**
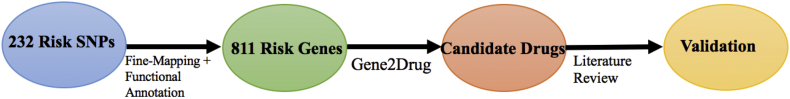
Flow chart of our methodology. Risk SNPs identified in previous IBD GWAS were fine mapped to 811 risk genes. Gene2Drug analysis was performed on the 811 risk genes, which yielded candidate drugs. To validate the candidates, the literature was then consulted to determine the most promising candidates.

Our analysis strategy to repurpose drugs for IBD using genetic information includes three steps (Fig. 1): mapping IBD risk genes from IBD risk variants, identifying the IBD candidate drugs using the risk genes as targets using Gene2Drug algorithm and validating the identified IBD candidate drugs through literature review. Below we will illustrate these three steps in details.

### 2.1 Mapping IBD Risk Genes from IBD Risk Variants

For the 232 IBD-associated SNPs identified in the latest meta-analysis of IBD GWAS [6,11], IBD risk genes were obtained using fine-mapping and functional annotation. First, for each IBD risk locus identified in the GWAS, the most likely causal SNP was identified using the Bayes factor calculated for each variant from the GWAS meta-analysis [6]. The Bayes factors were used to calculate the posterior probability that each variant is causal for a given region. These risk loci were defined by performing linkage disequilibrium (LD) regression to compute LD windows that define a locus. For every genome wide significant variant, the LD window was defined as the leftmost and rightmost variants that were correlated with the main variant with an r^2^ value of at least 0.6 [6]. Thus, for each risk locus computed, a causal variant was identified and the implicated variant would have the highest calculated posterior probability of all the variants in a given risk locus. The genes that the identified causal variants can be linked to were found using eQTL analysis, or targeted sequencing studies. For identifying eQTL overlaps, twelve eQTL were searched to identify variants within the identified risk loci that are associated with variation in gene expression [6]. The most significant variant-gene associations from each eQTL set were considered candidates if the variant had r^2^ > 0.8 with any of the lead SNPs in the IBD risk loci [6].

### 2.2 Identifying the IBD Candidate Drugs Using the Risk Genes as Targets

Once the risk IBD genes are mapped from the SNPs, drugs that significantly target these genes are desired. An online tool developed by Napolitano et al. [12] called “Gene2Drug” aims to find drugs that induce significant transcriptional modulation of pathways that involve a target gene of interest. The tool basically quantifies the drug-induced modulation of pathways that a gene of interest is involved in. The authors have developed an R package for the tool which was used to conduct the analysis [13]. The tool takes advantage of the procedure known as gene set enrichment analysis (GSEA) [14]. GSEA is a method that uses the ranked list of differentially expressed genes obtained from a GWAS to determine whether genes in a gene set of interest are significantly enriched at the top or bottom of the ranked list. For our purposes, we are comparing the ranked gene expression profiles between a control and drug treatment group to determine if a given gene set is significantly enriched in the drug treatment group. A gene set is a group of genes that are related through having similar biological functions, being located in the same region of a chromosome, or have similar regulation. This method is useful in identifying gene sets that are implicated in different disease phenotypes. Instead of looking at drugs that target gene sets, Gene2Drug is focused on identifying drugs that target pathway sets that an IBD risk gene of interest is involved in, using a variation of GSEA called pathway set enrichment analysis (PSEA). This was performed using 6 different pathway databases used as references. This approach will yield drugs that directly target the risk gene of interest while also targeting proteins that interact with the target, making this pathway based approach more comprehensive than traditional gene oriented ones. The obtained IBD genes are inputted into Gene2Drug to yield IBD candidate drugs. Gene2Drug uses built in gene expression data from the Connectivity Map (CMap) [15]. The CMap contains gene expression profiles of 5 cell lines treated with 1309 different small molecules or drugs. So for a given drug, the CMap tells us what genes are upregulated, downregulated, or unchanged in expression. Gene2Drug converts gene expression profile data from CMap into pathway expression profiles in order to perform PSEA. In this pathway-based CMap, pathways are ranked according to how much the expression of genes annotated to each pathway changes after drug treatment. Four steps were made to develop the Gene2Drug algorithm. First, Raw gene expression values from CMap are loaded; second, control-treatment fold change values are calculated for each drug-gene pair in CMap and a ranked drug-gene matrix is generated; third, given a pathway database used as reference, the genes in the matrix are converted to pathways using GSEA. In the final step, using the computed pathway-drug enrichment scores and p-values from the GSEA, a ranked pathway-drug matrix is generated [12]. Table 2 lists the six different pathway databases used for the Gene2Drug analysis. Multiple different pathway databases were used to ensure that the final candidate drugs identified targeted as many pathways as possible that the risk genes were involved in. For a single gene given as input, Gene2Drug finds all of the pathways that gene is involved in using a reference database. Then, using this subset of pathways that the inputted gene is involved in, PSEA calculates an Enrichment Score (ES) and a p-value for all 1309 CMap drugs. The p-value assesses the significance of the modulation of a pathway by a given drug. Computed p-values were adjusted using the Benjamini-Hochberg procedure to account for the high false positive rate associated with multiple testing procedures. The ES is a value that ranges from −1 to 1 that represents the degree in which a pathway is upregulated or downregulated by a given drug. Drugs at the top of the list are predicted to significantly activate the target, and drugs at the bottom are predicted to inhibit the target. PSEA calculates the ES using a generalization of the Kolmogorov-Smirnov statistic that assesses how much a set of pathways is towards the top or bottom of a ranked list of pathways [12].

**Table 2:** Pathway databases used for Gene2Drug analysis.

Source	Name	Description
MSigDB	GO BP	Gene Ontology – Biological Processes
MSigDB	GO MF	Gene Ontology – Molecular Function
MSigDB	GO CC	Gene Ontology – Cellular Component
MSigDB	CP	Expert-defined Canonical Pathways
MSigDB	C7	Immunologic Signatures
MSigDB	CM	Cancer Modules

Since six pathway databases were used, six different ranked drug lists were generated. For a drug to be considered as a candidate for literature review, it had to meet the false discovery rate cut-off of 0.01, and has appeared in all six drug lists after removing drugs with adjusted p-values >.01. A drug that appears in all six lists was desired because this drug would be likely to significantly target a large number of pathways that the IBD risk genes are involved in.

The Gene2Drug package used to perform this analysis was downloaded from https://bioconductor.org/packages/release/bioc/html/gep2pep.html. Default parameters were used when running this package.

### 2.3 Validating the Identified IBD Candidate Drugs

Once a list of candidate drugs was obtained, a literature review was performed to validate the results. The analysis performed to obtain the candidate drug list was only focused on the interactions between drugs, the risk genes, and the pathways the genes were involved in. Validation was required to ensure that the drug actually produces the desired interaction intended in studies done on that particular drug. It is also necessary to perform a literature review to make sure that the candidate drugs did not have any undesired side effects or were shown to be completely ineffective in studies or clinical trials. Validation was performed using PubMed (www.pubmed.gov) and repoDB (http://apps.chiragjpgroup.org/repoDB). PubMed was searched to find any studies that have been done on the candidate drugs regarding IBD. Any studies testing the efficacy or toxicity of the drugs were also of interest. It is thus desirable to find out whether clinical trials were undergone for the candidate IBD drugs to see if they are safe to use. The repoDB was consulted for this. The database contains data on drugs that have been looked at for repurposing. The database extracts data from ClinicalTrials.gov which has data on all drug clinical trials performed. The tool was used to see if the IBD candidate drugs have been looked at for repurposing in other diseases and to see if they had successfully passed those trials. When searching PubMed, the name of a given drug was included in the search along with key words such as “Inflammatory Bowel Disease”, “Crohn's Disease”, “Ulcerative Colitis” “inflammation”, “toxicity”, or “autoimmune”. In repoDB, the name of the drug of interest was the key search term.

## 3 Results

For the 232 IBD risk SNPs identified in GWAS meta-analyses, 811 risk genes were prioritized [6]. With these 811 risk genes, Gene2Drug analysis was performed using the six pathway databases. Using the described prioritization method, eight candidate drugs were obtained. Two of these drugs, Cephaeline and Helveticoside were excluded from the analysis due to the lack of information found on these drugs based on searches in PubMed and repoDB. This left six candidate IBD drugs, which are highlighted in Table 3, Table 4.

**Table 3:** List of candidate drugs obtained from Gene2Drug analysis and their link to IBD.

Drug	Link to IBD	Toxicity
Cefadroxil	• Alanine aminopeptidase (ANPEP): Involved in the production and processing of pro-inflammatory cytokines (IFN-*γ*, IL-1_ß_, IL-6, IL-8) [16] • Peptide Transporter 1 (PEP1): Abnormally upregulated in colon of IBD patients; induces inflammation through uptake of bacterial peptides from commensal bacteria [17] • Endothelin-1 Receptor (EDNRA): Enhances inflammation through recruitment of T lymphocytes and neutrophils, and release of pro-inflammatory and profibrotic cytokines [18]. • Pregnane X Receptor (NR1l2): Shown to protect against IBD through dysregulation of the nuclear factor-κB signaling cascade [19]	• Mild Side effects (nausea, vomiting, diarrhea, abdominal pain may occur) [26]
Propantheline Bromide	• Muscarinic acetylcholine receptor m1 (CHRM1): Muscarinic receptor antagonist that acts as a antispasmodic agent; not suitable for IBD overall [20]	• Mild side effects such as dry mouth, constipation and urinary retention [27] • Severe side effects such as tachyarrhythmia, hallucinations, delirium, and cognitive impairment can occur [27]
Lanatoside C	• Protein kinase C∂ (PRKCD): Involved in the regulation of several key IBD signaling pathways such as MAPK, NF-κB, c-Myc, and TNF-*α* [21]	• Can cause anorexia, nausea, vomiting, and neurological symptoms [28] • Can rarely trigger fatal arrhythmias [28]
Rifabutin	• Hsp90 (HSP90AA1, HSP90B1): downregulates pro-inflammatory cytokines IL-1b, IFN-*γ* and TNF-*α* [22]	• Mild side effects such as uveitis, rash, nausea, vomiting, neutropenia, anemia, discoloration of skin and body fluids [29] • Liver impairment (rarely) [29]
Dimethyloxalylglycine (DMOG)	• Increases NF-κB and HIF activity leading to enhanced epithelial barrier function through acting on Prolyl hydroxylases (EGLN1, EGLN2, EGLN3) [2] • Suppresses TGF-ß1 signaling reducing inflammation induced intestinal fibrosis [23]	• Increased erythropoiesis and angiogenesis [30]
Amphotericin B	• Transglutaminase 2 (TGM2): Activates NF-κB and promotes interleukin-6 expression [24] • Pyruvate carboxylase (PC): Positively regulates production of pro-inflammatory cytokines (IL-6, IL-8, IL-1ß, TNF-*α*) [25]	• Common side effects include chills, fever nausea, vomiting, and nephrotoxicity (can be fatal) [31]

**Table 4:** Ranking of candidate drugs in each database tested. Ranking was based on the computed enrichment scores and p-values for the candidate drugs for each database. P-values adjusted using the Benjamini-Hochberg Procedure.

Drug	Cefadroxil		Propantheline Bromide		Lanatoside C		Rifabutin		DMOG		Amphotericin B	
	Rank	P-Value	Rank	P-Value	Rank	P-Value	Rank	P-Value	Rank	P-Value	Rank	P-Value
GO: BP	409	8.47E-06	371	2.76E-06	362	9.20E-06	263	1.03E-06	262	4.09E-06	29	1.05E-08
GO: MF	11	2.63E-06	142	8.25E-04	76	9.77E-04	120	2.93E-03	30	4.92E-08	949	5.13E-04
GO: CC	135	4.19E-04	262	4.12E-03	205	9.99E-04	118	1.45E-04	206	5.37E-03	379	7.47E-03
CP	675	7.57E-03	668	6.42E-03	139	7.31E-05	186	7.31E-05	225	7.57E-03	24	1.31E-03
C7	14	4.70E-03	3	2.58E-04	101	3.10E-03	121	4.45E-03	91	4.28E-03	148	7.66E-03
CM	321	1.59E-04	454	2.36E-06	52	2.08E-04	32	7.25E-05	181	3.06E-03	63	1.20E-03

To find a link to IBD for each drug, the proteins these drugs target were looked at in the literature review (unless a study was found that linked the drug directly to IBD). Four of the six candidates have been successfully repositioned for other diseases based on clinical trial data (Table 5). These drugs would thus be considered safe to use in humans.

**Table 5:** Different diseases IBD candidate drugs have been repositioned for based on clinical trial data.

Drug	Disease tested for [7]	Clinical Trial Status [1]
Cefadroxil	Urinary Tract Infection	Approved
	Streptococcus Pyogens Infection	Approved
	Tonsillitis	Approved
	*Staphylococcus aureus* Infection	Approved
	Klebsiella Cystitis	Approved
Propantheline Bromide	Peptic Ulcer	Approved
Lanatoside C	None	
Rifabutin	*Mycobacterium* Avium Complex	Approved
	Infection	
	*Helicobacter Pylori* Infection	Approved
Dimethyloxalylglycine	None	
Amphotericin B	Bronchitis	Approved
	Psittacosis	Approved
	Sinusitis	Approved
	Pulmonary Cryptococcosis	Approved
	Syphilis	Approved
	Pneumonia	Approved

The two other drugs, lanatoside C and DMOG have been tested on human cell lines and animal models to good effect [30,32,33]. However, clinical trials would have to be performed on the drugs to ensure that they are suitable for humans.

**Fig. 2:**
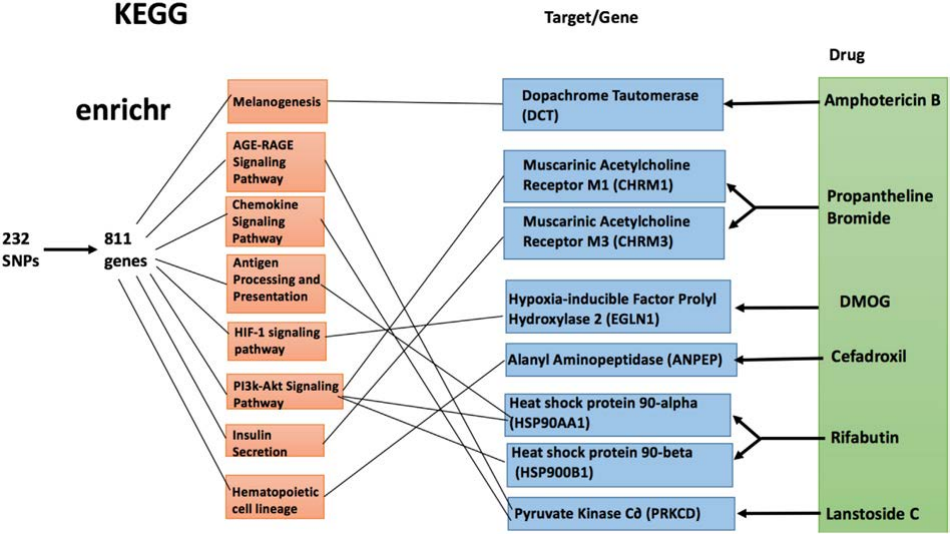
Enrichr analysis of the 811 IBD risk genes using KEGG. Pathways that met the adjusted p-value cutoff of 0.01 were considered significantly enriched in the 811 risk IBD genes. 44 KEGG pathways were found to be significantly enriched in the IBD risk genes.

**Fig. 3:**
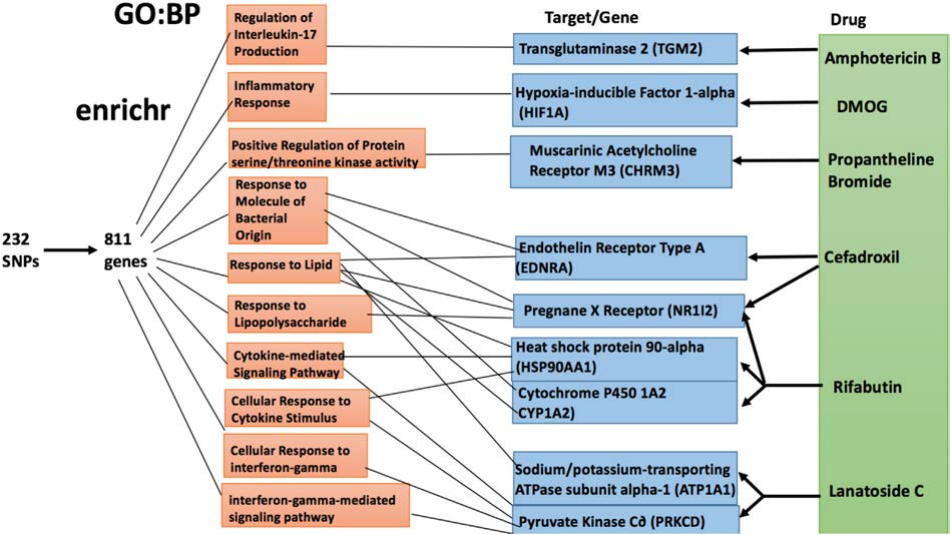
Enrichr analysis of the 811 IBD risk genes using GO:BP. GO terms that met the adjusted p-value cutoff of 0.01 were considered significantly enriched in the 811 risk IBD genes. 24 BP terms were found to be significantly enriched in the IBD risk genes.

For further validation, the gene set enrichment analysis tool known as Enrichr [34,35] was used. Enrichr analysis was performed on the 811 IBD risk genes and on genes that the six candidate drugs are known to target. This was done to determine whether there are enriched GO terms/pathways for the 811 risk genes that the drug target genes are involved in. If overlap occurred between at least one target of each candidate drug and the enriched sets of the risk genes, this would help confirm that the drugs actually target these risk genes. The results from the Enrichr analysis are depicted in Fig. 2, Fig. 3. Enrichr analysis was performed on the two gene sets using the Biological Processes (BP) gene ontology and the Kyoto Encyclopedia of Genes and Genomes (KEGG) pathway database. The two Enrichr analyses performed indicate that the risk genes and drug targets do in fact overlap in biological processes and KEGG pathway. This fact helps validate the candidate drugs chosen as it provides evidence that the drugs interact with the risk genes.

## 4 Discussion

Cefadroxil is a first generation cephalosporin antibiotic that is primarily used to treat urinary tract infections, skin infections, pharyngitis, and tonsillitis. Side effects are mild, and the drug has low toxicity [36]. Although the drug is primarily concerned with bacterial infections, it targets interesting human proteins in relation to IBD.

One target of the drug is the enzyme alanine aminopeptidase [37]. The enzyme is a zinc dependent endopeptidase that degrades proteins with a neutrally charged N-terminal amino acid. The enzyme has many functions, including regulation of peptides, tumor cell invasion, differentiation, proliferation, apoptosis, motility, and angiogenesis [38]. The enzyme has been looked at as a target for cancer chemotherapy due to its role in malignant cell regulation. A link has also been made between the enzyme and IBD. IBD is characterized by dysregulation of certain subsets of T cells. These subsets of T cells express the aminopeptidase enzyme and are used to control immune response through hydrolyzing bioactive peptides [39]. Some activated T cells show surface expression of the enzyme, which is believed to increase production of pro-inflammatory cytokines. This illustrates why the enzyme has been looked at as a target for IBD therapy. Inhibition of the enzyme could decrease the over active immune response associated with IBD and reduce discomfort in some cases. Cefadroxil inhibits alanine aminopeptidase which would support its use as an IBD drug. The enzyme is also responsible for the formation of proinflammatory cytokines such as leukotriene B_4_ and leukotriene A_4_ [16]. A study by Bank et al. [39], on the potential of aminopeptidase inhibitors for IBD treatment concluded that the protein was an interesting target. They also concluded that the functions of the protein need to be understood, and that they were unsure if the inhibitors are replicable in humans (mouse models were used).

Another interesting target of cefadroxil is peptide transporter 1 (PepT1) [36]. Primarily expressed in the small intestine, the protein transports dipeptides and tripeptides into the small intestine for absorption. Upregulation of the protein in the colon has been seen in those suffering from chronic inflammation. In healthy individuals, the expression of this protein in the colon is low. Upregulation of the protein in the colon causes the transport of bacterial peptides into the colon cell which triggers proinflammatory immune responses once the bacterial peptides interact with innate immune system cells [17]. The protein is thus associated with chronic inflammation and is a possible target for drugs that reduce IBD symptoms. The protein is also an appealing drug target due to its ability to transport peptide-derived drugs, meaning it can be delivered orally since the protein is mainly expressed in the small intestine [17]. Cefadroxil inhibits PepT1, further reinforcing its candidacy for drug repositioning.

Cefadroxil also targets the endothelin-1 receptor, mediates vasoconstriction in endothelial cells of blood vessels upon endothelin-1 binding [36]. Excessive production of endothelin-1 in the GI tract due to immune system activation causes dysregulation of cytokine production. Endothelin-1, produced by endothelial cells, is important in inflammation where they recruit circulating leukocytes to the endothelium [18]. Overproduction of Endothelin-1 is thus associated with excessive inflammation that characterizes IBD. Endothelin-1 has also been shown to induce mast cell degranulation. Excessive degranulation of mast cells causes damage to intestinal mucosa. Studies have shown an increased presence of endothelin-1 in tissues from patients with IBD [18]. It has been suggested that vasoconstriction induced by the molecule makes it act as a chemoattractant which exacerbates tissue damage in IBD due to inappropriate inflammation [18]. Thus, reducing endothelin-1 activity through blocking endothelin-1 receptors appears to be a potential strategy in IBD drug development. Studies investigating the potential of endothelin-1 antagonists have shown potential as anti-inflammatory agents [18]. Cefadroxil acts as an endothelin-1 receptor antagonist, meaning it affects another target associated with IBD.

Cefadroxil also targets the pregnane X receptor (PXR), a nuclear receptor that controls genes involved in drug transport and detoxification [37]. PXR aids in protection against IBD through downregulation of the nuclear factor-κB (NF-κB) signaling cascade [19]. This cascade regulates many cellular functions including DNA transcription, immune system response (innate immune system and inflammation), and apoptosis. Its role in inducing pro-inflammatory gene transcription makes it a target to drugs dealing with inflammatory disorders such as IBD [40]. This illustrates the role of PXR in protecting from IBD; downregulation of the NF-κB cascade decreases inflammation, and T cell proliferation [40]. This has led to clinical trials, which have shown promise in the use of PXR agonist drugs for IBD. Although cefadroxil is a PXR agonist, it has been shown to only weakly enhance the protein's activity [41].

Propantheline bromide is an anticholinergic drug commonly used to treat enuresis, hyperhidrosis, and spasms of the stomach, intestines or bladder [36]. It is a muscarinic receptor antagonist that reduces stimulation of muscles by preventing acetylcholine released by nerves from binding to muscarinic receptors. The drug is thus an antispasmodic that relieves pain caused by smooth muscle spasms in the gut. Antispasmodics would appear to be useful in IBD treatments as they would bring pain relief from the intestinal spasms associated with the disorder. Studies investigating the use of antispasmodics have been found to relieve pain due to inflammation but overall, their efficacy in treating IBD overall is low [20]. The side effects of antispasmodics, mainly severe obstruction in a partially obstructed gut, and decreased motility are further problems with using them in IBD treatments. There have been no studies testing the use of propantheline bromide in IBD. The drug could help reduce pain from inflammation in patients, but the long term use of the drug is unlikely.

Lanatoside C is a cardiac glycoside commonly used to treat congestive heart failure and cardiac arrhythmia [42]. This function is due to targeting of the sodium-potassium ATPase, which disrupts movement of sodium and potassium across the cell membrane. The drug is being investigated for use in chemotherapy due to its inhibition of cancer cell proliferation and migration, and its induction of cell cycle arrest and apoptosis in cancer cells [42]. It is currently thought that the drug exerts these anti-cancer activities through inhibition of the Wnt signaling pathway, which is involved in many cell processes, such as cell proliferation, the cell cycle, and cell invasion. The apoptosis inducing effects of the drug could make it a possible candidate for IBD treatment. Chronic inflammation, which is a main symptom of IBD is due partly to T lymphocytes in the lamina propria that are resistant to apoptosis [43]. If the drug can induce apoptosis in the resistant T lymphocytes as effectively as it has shown to in cancer cells, then the drug could be of use for IBD. Lanatoside C has also been found to activate protein kinase C∂ [44]. The protein kinases are thought to play a significant role in IBD, and are considered potential targets against the disease [21]. This is due to their involvement in many signaling pathways that control inflammation, proliferation, and tumorigenesis. Along with the NF-κB signaling cascade that was previously discussed, protein kinases are also involved with the MAPK signaling pathway [21]. This pathway is heavily associated with IBD, where it upregulates pro-inflammatory protein and immune cell activity. Protein kinase C∂ is involved in many cellular processes, including regulation of cell growth and apoptosis. The pro apoptotic effects of the protein are believed to occur through activation by caspase-3, which cleaves the kinase and releases its catalytic domain into the mitochondria, triggering the intrinsic pathway of apoptosis. Lanatoside C induces caspase-3 and thus protein kinase C∂ activity making it an activator of apoptosis. Protein kinase C∂ can also trigger apoptosis through the extrinsic pathway by translocating to the cell membrane and modulating the secretion of tumor necrosis factor-*α*, a crucial signaling molecule in IBD that induces apoptosis [45]. This is mediated by caspase-8; lanatoside C has been shown to activate this apoptotic pathway through protein kinase C∂ as well [44]. The benefits of increased apoptosis activation towards reducing IBD inflammation have been described previously. The pro-apoptotic effects of lanatoside C make it an interesting drug in relation to IBD. However, the drug has primarily been looked as a candidate for repositioning in cancer, and there have been no studies that have investigated the use of the drug for IBD.

Rifabutin is a broad-spectrum antibiotic used for the prevention of disseminated *Mycobacterium avium* complex disease in those with an advanced HIV infection [36]. The drug has also been shown to be effective in treating multidrug-resistant*Helicobacter pylori* [46]. Rifabutin targets the heat shock protein 90 (Hsp90), a molecular chaperone activated by cellular stress that stabilizes signaling proteins involved in cell growth and survival [47]. There are two types of the protein: Hsp90*α*, involved in cell proliferation; Hsp90ß, involved in cell differentiation and structure formation. Rifabutin targets both types of Hsp90 [37]. The protein is clinically associated with cancer, due to the finding that it is expressed at a greater level in tumor cells. Hsp90 is involved in regulating tumor cell proliferation, survival, invasion, metastasis, angiogenesis, and other processes [47]. Hsp90 also has an important role in inflammation, making it important with regards to IBD. During inflammation, the protein represses heat-shock factor 1, which causes the activation of pro-inflammatory proteins and inhibition of anti-inflammatory proteins [22]. The protein is also involved in NF-κB signaling, and it upregulates TNF--*α*. Hsp90; therefore, has been identified as a potential target for IBD drug development. Rifabutin is thus an interesting drug in regards to IBD due to its inhibition of Hsp 90, which is a part of many important cellular pathways involved in the disease.

**Fig. 4:**
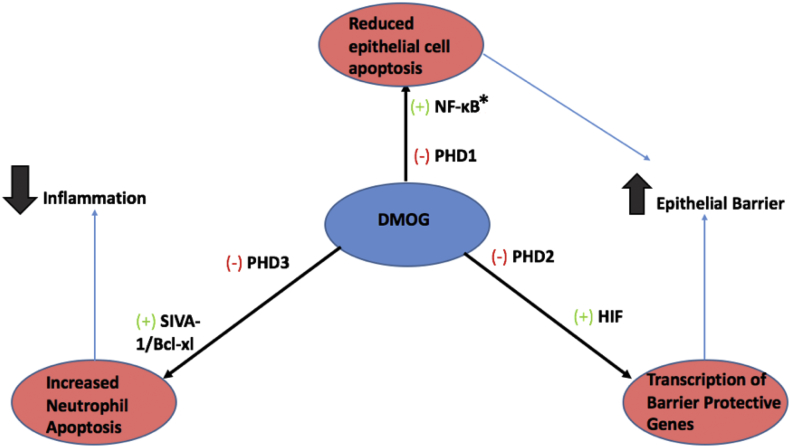
Mechanism of DMOG in reducing intestinal inflammation. The drug acts through the inhibition of three hydroxylases. Inhibition of PHD1 leads to reduced epithelial cell apoptosis. Inhibition of PHD2 leads to increased transcription of barrier protective genes which further strengthens the epithelial barrier. Inhibition of PHD3 leads to increased neutrophil apoptosis leading to decreased inflammation.

Dimethyloxalylglycine (DMOG) is a hydroxylase inhibitor that has shown potential to be a candidate IBD drug [30,32]. Hydroxylases are oxygen-sensing enzymes which confer oxygen-dependence onto hypoxia-sensitive transcriptional regulators such as HIF and NF- κB [30,32]. The expression of these transcription factors are believed to be deregulated in chronic inflammatory disorders such as IBD. Hypoxia is a key feature of IBD caused by high oxygen consumption of inflamed tissues, infiltrating immunocytes, and vascular dysfunction [48]. As mentioned previously, NF- κB is a master regulator of innate immunity, inflammation, and apoptosis making it highly significant with respect to IBD. HIF is a transcription factor induced by hydroxylases in hypoxic conditions that up-regulates metabolic enzymes, angiogenic factors, and vasoactive substances which increase oxygen supply and metabolic activity in the hypoxic tissue [48]. The role of the HIF pathway in immune system regulations is not completely understood. Studies have found that the pathway is linked to innate and adaptive immune system regulation, and inflammation [48]. The activity of the NF-κB pathway is upregulated in hypoxic conditions. It is thus believed that there is cross-talk between the NF- κB and HIF pathways. This suggests that modulation of the HIF pathway can indirectly modulate the NF- κB pathway and thus regulate immune system activity in tissues. This has been illustrated by studies investigating the use of DMOG, a hydroxylase inhibitor in reducing intestinal inflammation. Cummins et al. [2], found DMOG to have therapeutic effects towards IBD through enhancing HIF and NF- κB activity. The drug improves epithelial cell barrier function through inhibition of prolyl hydrosylase 1 (PHD), which leads to increased NF- κB signaling, and reduced epithelial cell apoptosis. Inhibition of prolyl hydroxylase 2 (PHD 2) increases HIF activity, which leads to activation of barrier protective genes in intestinal epithelial cells. This barrier is essential in protecting the intestines from inflammatory triggers such as bacteria and viruses. A damaged barrier will allow bacteria from the microbiome to leak into the intestines and trigger inflammation. Finally, inhibition of neutrophil prolyl hydroxylase 3, another hypoxia response regulator by DMOG, leads to enhanced neutrophil apoptosis and decreased inflammatory activity [2]. The overall mechanism of DMOG in reducing intestinal inflammation was adapted from [2], and depicted in Fig. 4.

**Fig. 5:**
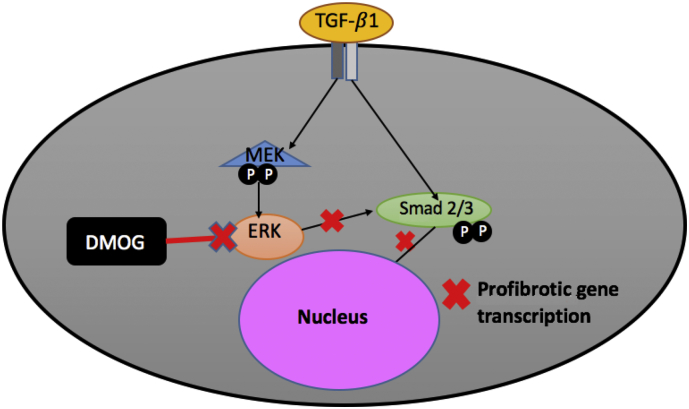
Mechanism of TGF-ß1 signaling inhibition by DMOG. DMOG reduces ERK and Smad2/3 cross-talk. Smad 2/3 is a transcription factor that induces transcription of profibrotic genes. Decreased rates of Smad 2/3 phosphorylation by ERK leads to decreased transcription of these genes and decreased fibrosis.

DMOG has also shown efficacy in treating intestinal fibrosis, the excess formation of scar tissue that can be a complication of IBD. Fibrosis is the result of an overactive wound healing response that results in excessive deposition of extracellular matrix; excessive fibrosis leads to interference in organ functioning. Studies have found DMOG to reduce intestinal fibrosis in addition to its anti-inflammatory effects [30,32]. This inhibition is believed to occur through reducing transforming growth factor-ß1 (TGF-ß1) mediated activation of ERK signaling pathway, which activates intestinal fibroblasts [23]. TGF-ß1 and ERK are part of the MAP-Kinase signaling pathway, which plays an important role in the regulation of cell growth, proliferation, and differentiation. The overall mechanism of the drug on this pathway was proposed by Manresa et al., [23], and is illustrated in Fig. 5.

In terms of side effects, DMG has shown to be well tolerated in murine models with collitis [30,32]. Problematic side effects include erythropoiesis and angiogenesis, which would reduce the usefulness of systemic hydroxylase inhibitors [30,32]. Delivery of the drug is also a concern, with oral administration of the drug ineffective due to inactivation by stomach acid [30,32]. The main objective to determine whether the drug is suitable for IBD would be testing the drug on humans in clinical trials. Overall, DMOG was found to be the most promising candidate for IBD based on studies performed on the drug.

Amphotericin B is a broad-spectrum polyene macrolide antibiotic used for serious fungal infections such as mycosis [49]. The drug has not been investigated for therapeutic potential in IBD, but targets some proteins with regards to IBD. The drug upregulates dopachrome tautomerase (DCT), a protein that mimics the effects of macrophage migration inhibitory factor (MIF) [50]. MIF is a pro-inflammatory protein that plays a significant role in inflammatory pathways, and has been implicated in many cancer phenotypes [50]. It is likely that Amphotericin B would therefore worsen IBD symptoms through DCT. Amphotericin B has also been shown to inhibit transglutimase 2 (TG2), a calcium dependent enzyme that catalyzes crosslinking, polyamination or deamidation of glutamine residues in proteins [24,51]. The protein is linked to many inflammatory diseases, including celiac disease, pulmonary fibrosis, cystic fibrosis, multiple sclerosis and sepsis [24]. TG2 is activated by oxidative stress caused by tissue injury, inflammation or hypoxia. When activated, the protein covalently activates many proteins that results in modulation of cell adhesion, cytokine production and cell survival [24]. TG2 thus has a role in triggering inflammation and so down regulating its activity would likely be useful in treatment in IBD. Studies have found significantly high levels of TG2 antibodies detected in the serum of IBD patients, further suggesting the protein is active in the pathogenesis of IBD (Likely through inducing Th17 cell differentiation) [24]. Amphotericin C has shown to inhibit pyruvate carboxylase (PC) activity in the mitochondria [52]. PC is part of the biotin-containing enzyme family that plays a role in many biological processes, such as fatty acid synthesis, glyceroneogenesis, and glutamate production [25]. The protein is also involved in the antiviral immune response, where it activates NF- κB, enhances production of type 1 interferons and pro-inflammatory cytokines [25]. The ability of PC to up-regulate inflammation would make it appear to be a potential IBD target, but the fact that these effects are only induced upon virus detection, make it an unsuitable target for IBD. Amphotericin B is also limited by its toxicity. The drug is toxic to normal tissues; side effects include infusion-related toxicity and chronic toxicities such as nephrotoxicity, hemolysis, cardiotoxicity, hepatoxicity, leukopenia and thrombocytopenia [49]. Overall, amphotericin B would be unlikely to be a candidate for repurposing towards IBD. Reasons for this include its toxicity and unsuitable protein targets. While TG2 is a suitable target, upregulation of DCT would be detrimental to IBD treatments, while PC activation would be unlikely to have any effect in IBD patients.

## 5 Conclusion

The high quality genetic data collected for IBD over the past few years was taken advantage of to find potential candidate drugs that could be repurposed or developed for the disorder. Six candidate drugs were obtained. The results were validated by showing that the drug targets of these candidate drugs are involved in common biological processes and pathways with the 811 prioritized risk genes. The drugs were further validated by checking to see if they had been involved in clinical trials or animal studies. The most promising candidate was DMOG due to the findings that it can reduce intestinal inflammation and fibrosis in animal models. Further validation of the drug is required. No studies have looked at the drug in humans.

There have been IBD drug repositioning studies performed in the past that share similarities with, but are ultimately different to the one conducted. Clark et al. [53], used large scale data, bioinformatics tools, and high-throughput computations to obtain gene and microRNA signatures for CD and UC. They used these signatures to obtain genes that could be potential drug targets through consultation of literature. In contrast to our analysis, gene expression levels were looked at through microarray and microRNA analysis to find genes with significantly altered expression in IBD patients [53]. Our study had a much more comprehensive set of data to work with; the GWAS performed by de Lange et al. [6] had a total of 12,160 IBD cases while the study performed by Clark et al. [53] only had access to 118 cases. Our study was also conducted at a time where the underlying genetics of IBD were better understood and thus had access to a larger and more accurate source of IBD genetic variant data. This would give us a more accurate risk gene list that provided better targets for the drug repositioning analysis.

Another IBD drug repositioning study performed by Cai et al. [54] used gene expression data to find potential IBD drug candidates. They developed a drug repositioning framework through comparing IBD genomic profiles with drug induced genomic profiles to find matches [54]. The most promising drugs from this analysis were found to be Naloxone and Naltrexone, which both showed potential in treating IBD [54]. The advantage of our study is the integration of pathway data as opposed to just the genetic information in the drug repositioning analysis. This allows for the finding of candidate drugs that indirectly target a given risk gene; our approach is more layered, allowing for more potential drug candidates.

A IBD drug repositioning study conducted by Collij et al. [9] performed a protein-protein interaction analysis rather than a pathway based approach. They used IBD risk SNPs identified in GWAS, compiled a list of risk genes from this information, and then identified drugs that targeted the proteins encoded by these genes [9]. Their approach yielded 113 potential IBD drugs; 14 are used in treatment of IBD already, 48 have been investigated for IBD use, 19 have been looked at for other inflammatory disorders, and 32 had working mechanisms that were deemed interesting [9]. The study lacked a method of ranking the drugs they obtained in the results, no top candidates were identified. While the study looked at drugs that can target proteins of risk IBD genes and genes that interact directly with these genes, our study is able to identify drugs that target proteins that directly or indirectly interact with risk genes identified.

Very few studies have used the IBD genetic data obtained from GWAS for clinical purposes, making this sort of drug repurposing study useful. Development of new IBD drugs is highly desirable due to the side effects and lack of efficacy over time of current IBD drugs. The high costs of developing drugs also mean more efficient methods of development are required. The use of genetic information to repurpose drugs could also allow clinicians bypass the dilemma in choosing between the standard top-down and step-up treatment approaches; personalized medicine based on a patient's genome would allow the use of drugs based on the patient's genetic composition and disease course.

There are limitations to our approach. The databases used for this study are incomplete in terms of information linking identified risk genes to drugs. The biology of these risk genes and the genes targeted by drugs needs further investigation on what genes and drugs to focus on for the identification of suitable treatment drugs. The lack of information regarding the genes and drug targets was illustrated by the fact that two potential candidate drugs; helveticoside and cephaeline, had to be excluded due to the lack of data available on the mechanisms of these drugs and the genes they target.

Our study provided a preliminary look at the use of genetic information in drug repurposing for IBD. While this study cannot be conclusive, six potential candidate drugs for IBD were obtained. Further validation of the candidates is necessary and this will be aided by the continuously increasing information on genes, drugs, and the proteins they target.

## Conflicts of Interest

The authors declare no conflict of interest.

## Funding

This work was supported in part by The Children’s Hospital Foundation of Manitoba, Natural Sciences and Engineering Research Council of Canada, Manitoba Health Research Council, Health Sciences Centre Foundation, Mitac and University of Manitoba.
